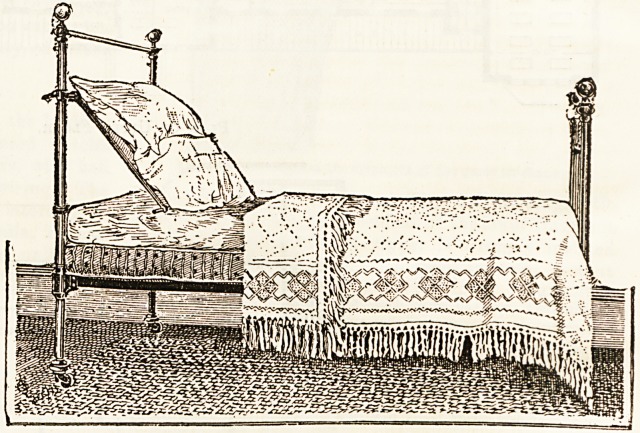# Practical Departments

**Published:** 1894-08-04

**Authors:** 


### PRACTICAL DEPARTMENTS.

### A NEW BEDSTEAD.

Inventions for the greater comfort and convenience of those who are compelled by ill-health to spend weary days and nights prostrate on their beds are ever on the increase, and a really enormous amount of ingenuity is expended nowadays on sick-room specialities. One which has recently come to our notice is shown in the accompanying drawing, and is intended to unite the usefulness of a bed-rest, and that an exceptionally firm and steady one, with the advantage of its entire disappearance when not actually needed.

It will be seen that it forms an integral part of the bedstead, being, indeed, the whole headrail cunningly made moveable and slipped into position with perfect ease. The bottom rail is lifted from the slots where it rests, and being run forward the rings slide down the pillars until the wished-for angle is obtained, when by turning a nut at the side the rest will remain secure and the pillows can be replaced. The change is made so easily that the occupant of the bed can manage it himself with little exertion, and any further lowering or raising can be conveniently carried out also.

The bedsteads are very light and pretty looking, and are made in several qualities at prices varying from £1 4s. 6d. to £8 5s. 6d.—the more expensive being all brass. The idea is the outcome of practical experience, the inventor, the Rev. W. Goodliffe, having been for many long months a helpless invalid.

The convenience of having the rest always at hand and ready for use, while when not needed it simply does not exist to take up room, will readily be appreciated. We should not omit another feature, which is that the headrail will slip right away down behind the bed if required. The bedsteads are manufactured by the "Cambridge" Bedstead Company, 5, Free School Lane, Cambridge, or they may be seen and procured at Messrs. Atkinson's, the well-known furniture firm in Westminster Bridge Road.

## Figures and Tables

**Figure f1:**